# Associations among Self-Reported Laryngopharyngeal Reflux, Allergic Symptoms, and Type of Dysphonia

**DOI:** 10.1055/s-0046-1818630

**Published:** 2026-04-30

**Authors:** Abdul-Latif Hamdan, Yara Yammine, Patrick Abou Raji Feghali, Jad Hosri, Lana Ghzayel, Jessica Aoun

**Affiliations:** 1 Department of Otolaryngology and Head & Neck Surgery, American University of Beirut-Medical Center, Beirut, Lebanon; 2 Department of Pathology and Laboratory Medicine, American University of Beirut-Medical Center, Beirut, Lebanon

**Keywords:** laryngopharyngeal reflux, allergy, dysphonia, voice, laryngology

## Abstract

**Introduction:**

Dysphonia is defined as a change in voice timber, pitch, or loudness. Precipitating factors may be occupational or health-related, such as allergy and laryngopharyngeal reflux (LPR).

**Objective:**

To investigate the association between combined LPR, and allergic rhinitis (AR), and type of dysphonia, that is, functional versus non-functional.

**Methods:**

A retrospective chart review of all patients with dysphonia who presented to the Voice Unit at a tertiary referral center between November 2018 and February 2022 was conducted. Patients must have previously filled out the Reflux Symptom Index (RSI) and AR questionnaires to be recruited. Based on laryngeal examination, the etiology of dysphonia was stratified as functional versus non-functional (referred to as structural/neurologic).

**Results:**

A total of 137 patients were included, 65 (47.4%) of whom had a structural voice disorder, 22 (16.1%) had neurologic voice disorders, and 50 (36.5%) had a functional voice disorder. Seventy-eight patients (56.1%) had a positive RSI ≥ 13, suggestive of LPR. In these patients, dysphonia was functional in 31, structural-in 34, and neurologic in 13 ( *p* = 0.568). Sixty-eight patients (48.9%) had a positive AR score ≥ 1, suggestive of allergy, 62% of whom had functional dysphonia, 46.2% had structural dysphonia, and 31.8% had neurologic dysphonia ( *p* = 0.046). Twenty-five of the 50 patients (50%) with functional dysphonia had RSI ≥ 13 and AR ≥ 1 versus 29.3% (19/65) of the structural subgroup and 31.8% (7/22) of the neurologic subgroup ( *p* = 0.063). After adjusting for smoking, patients with both reflux and allergy had 2.913 (95%CI = 1.32–6.442) times the odds having functional dysphonia compared with those having either or none ( *p* = 0.008).

**Conclusion:**

Patients with dysphonia and history of LPR and allergy are more likely to have a functional underlying etiology.

## Introduction

Dysphonia is defined as a change in voice timber, pitch, or loudness. 1 2 Precipitating factors may be occupational or health-related such as allergy and laryngopharyngeal reflux. Allergy is a public health concern affecting at least 20% of the population. 3 Numerous studies have shown that patients with allergic rhinitis (AR) have a higher prevalence of dysphonia than subjects without it. 4 5 6 Similarly, patients with dysphonia are more likely to have allergy than patients with no dysphonia. In a study on the incidence of AR in singers, Hamdan et al. noted that singers with dysphonia were 15% more likely to have AR than singers with no dysphonia. 7 The dysphonia is ascribed mostly to trafficking of mucus from the sino-nasal cavities into the larynx resulting in vocal fold inflammatory changes, including secondary edema of the vocal folds. 6 8 9

Laryngopharyngeal reflux (LPR) is also recognized as a risk factor for dysphonia. It is an extraesophageal variant of gastroesophageal reflux disease, and it is characterized by retrograde movement of gastroduodenal contents into the laryngopharyngeal complex resulting in mucosal inflammation. Patients with LPR often present with hoarseness, throat clearing, chronic cough, and globus pharyngeus. The consensus is that one of two patients with dysphonia may suffer from LPR. 10 11 12

Given that LPR and AR are independent risk factors for dysphonia, no other study investigated the role of both LPR and AR as comorbidities in patients with dysphonia. The authors of the present manuscript aim to investigate the possible association between combined LPR/AR and type of dysphonia, that is, functional or non-functional. Functional dysphonia is defined as dysphonia in the absence of identifiable structural and/or neurological disorder. The authors attempt to answer the following question: Are patients with dysphonia and history suggestive of LPR and AR more likely to have functional dysphonia in comparison to those with no LPR and AR?

## Methods

After having obtained the Institutional Review Board's approval (IRB ID: BIO-2022–0091), a retrospective chart review of all patients who presented to the Voice and Swallowing Unit at a tertiary referral center between November 2018 and February 2022 was conducted. Patients who presented with dysphonia as a chief complaint were included in the study, while patients with incomplete medical records were excluded. Dysphonia was defined as a change in timber, pitch, or loudness. 13 Only those whose electronic medical records included filled Reflux Symptom Index (RSI) 14 and AR questionnaires 15 were recruited. Since the current study is a retrospective chart review with no more than minimal risk, the IRB waived the requirement for informed consent. The RSI and AR questionnaires are routinely used for screening patients who present with dysphonia. Patients with incomplete medical records for any reason were excluded. History of LPR was suggested when the patient had a score above 13, and history of allergy was suggested when the patient had a score above 1. Demographic data included age, gender, smoking history, medical history, and vocal history.

Based on the laryngeal examination performed using the RLS 91008 rhino-laryngeal stroboscope (PENTAX Medical), the etiology of dysphonia was stratified as structural/neurologic, also referred to as non-functional, versus functional. Structural dysphonia was characterized by the presence of vocal fold mucosal lesions such as leukoplakia, papilloma, or exudative lesions of the lamina propria such as nodules, polyps, cysts, Reinke's edema, and fibrovascular masses. Neurologic dysphonia was characterized by the presence of vocal fold-impaired mobility, such as paresis or paralysis, laryngeal dystonia, tremor, and/or history of inducible laryngeal obstruction. Patients with no structural/neurologic etiology were categorized as having functional dysphonia. 16

### Statistical Method

Frequencies and mean ± standard deviation (SD) values were used to describe categorical and continuous variables, respectively. Categorical variables were analyzed using the Pearson χ ^2^ test, whereas continuous variables were analyzed using the Mann-Whitney U test after the Shapiro-Wilk normality test showed the variables to significantly deviate from normal distribution. The Pearson χ ^2^ test was used to analyze the difference in the categorical variables between two groups and to calculate the *p* -value. A two-tailed *p-* value of < 0.05 was considered statistically significant. Unadjusted and adjusted odds ratios were computed to measure the association between variables and 95% confidence interval for each odds ratio was computed.

To adjust for potential confounding factors, linear regression analyses were conducted for RSI and Allergic Rhinitis Questionnaire, with age, gender, and smoking status included as covariates with no significant associations between the demographic data with either outcome.

All analyses were conducted using Statistical Package for the Social Sciences (IBM Corp.) version 24.0 software.

## Results

### Demographic Data

A retrospective chart review of 1,280 charts was performed. A total of 137 patients who had completed both the RSI and the allergy screening questionnaires were included in the current study. These were divided into 74 male (54%) and 63 female subjects (46%). The mean age of the study population was 51.26 ± 17.16 years, with a minimum age of 16 and maximum of 85. Sixty-five (47.4%) had a structural voice disorder, 22 (16.1%) had neurologic voice disorders while 50 (36.5%) had a functional voice disorder. The mean age of patients with functional dysphonia was 48.54 ± 19.38 years compared with 52.34 ± 15.70 years for patients with structural dysphonia and 54.27 ± 15.75 years for patients with neurologic dysphonia ( *p* -value = 0.435). As for gender, the male-to-female ratio was 1:1, 3:2 and 5:6 for the functional, structural and neurologic groups, respectively ( *p* -value = 0.385). Among the patients with functional dysphonia, 44.0% were smokers, whereas among patients with structural dysphonia, 47.7% were smokers, and among patients with neurologic dysphonia, 36.4% were smokers ( *p* -value = 0.004) ( Table 1 ).

**Table 1:** Demographic data of the study population

	Functional (n = 50)	Structural (n = 65)	Neurological (n = 22)	*p* -value
Mean age (years)	48.54 ± 19.38	52.34 ± 15.70	54.27 ± 15.75	0.435
Gender (male:female)	1:1	3:2	5:6	0.385
Smoking (n [%])	22 (44.0)	31 (47.7%)	8 (36.4%)	0.004*

**Note** : *Statistically significant ( *p* -value < 0.05)

### Reflux Symptom Index Score in the Total Group and Subgroups

The mean RSI score of the total group was 13.92 ± 6.98. Seventy-eight patients out of the 137 (56.1%) had a positive score, RSI ≥ 13, suggestive of LPR. When stratified by etiology, 62% of patients with functional dysphonia (31/50) had positive RSI score compared with 52.3% (34/65) of patients with structural dysphonia and 59.1% (13/22) of patients with neurologic dysphonia. The difference in the prevalence of positive scores suggestive of LPR between the subgroups was not statistically significant ( *p* -value = 0.568). There was also no significant difference in the mean score of the RSI in patients with functional, structural, and neurologic dysphonia ( *p* -value = 0.308) ( Table 2 ).

**Table 2:** Prevalence of self-reported symptoms in functional versus structural dysphonia

	Functional (n = 50)	Structural (n = 65)	Neurological (n = 22)	*p* -value
Mean VHI-10	13.5 ± 8.1	13.83 ± 6.70	20.71 ± 8.32	0.002*
Mean RSI score	14.82 ± 7.50	12.80 ± 6.30	15.18 ± 7.43	0.308
< 13	19 (38%)	31 (47.6%)	9 (40.9%)	
≥ 13	31 (62%)	34 (52.3%)	13 (59%)	0.568
Mean AR score	0.94 ± 0.99	0.71 ± 0.89	0.45 ± 0.80	0.05*
< 1	19 (38%)	35 (53.8%)	15 (68.2%)	
≥ 1	31 (62%)	30 (46.2%)	7 (31.8%)	0.046*

**Abbreviation** : AR: allergic rhinitis; RSI: Reflux Symptom Index; VHI-10: Voice Handicap Index-10.

**Note** : *Statistically significant ( *p* -value < 0.05).

### Allergic Rhinitis Score in the Total Group and Subgroups

The mean allergic rhinitis (AR) score in the total group was 0.76 ± 0.93. Sixty-eight patients (48.9%) had a positive score, that is, AR ≥ 1, suggestive of allergy. Sixty-two percent of patients with functional dysphonia (31/50) had positive AR score compared with 46.2% (30/65) of patients with structural dysphonia and 31.8% (7/22) of patients with neurologic dysphonia. The difference between the subgroups was statistically significant ( *p* -value = 0.046). There was also a significant difference in the mean score of AR in patients with functional, structural, and neurologic dysphonia (mean rank = 77.42, 66.98 and 55.82, respectively) ( *p* -value = 0.05) ( Table 2 ).

### Reflux Symptom Index Score and Allergic Rhinitis Score in the Total Group and Subgroups

Fifty out of 137 patients included in this study had an RSI above 13 and a positive AR score, thus suggestive of having both LPR and AR. The prevalence of history of LPR and allergy based on the RSI and AR scores was found to be higher in patients with functional dysphonia as compared with patients with structural/neurologic dysphonia. Twenty-five of the 50 patients with functional dysphonia, that is, 50%, had an RSI above 13 and a positive AR score in comparison to 29.3% (19/65) of the structural subgroup and 31.8% (7/22) of the neurological subgroup. The difference between the subgroups was not statistically significant ( *p* -value = 0.063) ( Table 3 ).

**Table 3:** Prevalence of self-reported symptoms in functional versus structural dysphonia

Category	Functional pathology (n = 50)	Structural pathology (n = 65)	Neurological (n = 22)	*p* -value
**AR ≥ 1 and RSI ≥ 13**	**25 (50%)**	**19 (29.3%)**	**7 (31.8%)**	**0.063**
Other	25 (50%)	46 (70.7%)	15 (68.2%)	

**Abbreviations** : AR: allergic rhinitis; RSI: Reflux Symptom Index.

**Note** : *Statistically significant ( *p* -value < 0.05).

Patients with history suggestive of LPR and allergy had 2.33 (95%CI = 0.704–7.755) times the odds of having functional dysphonia compared with those having either or none. This difference was not statistically significant ( *p* -value = 0.166). After adjusting for smoking, patients with both reflux and allergy had 2.913 (95%CI = 1.32–6.442) times the odds of having functional dysphonia compared with those having either or none, that is, they were almost 3 times more likely to have no structural or neurologic laryngeal disorder on examination compared with those with no history suggestive of LPR and AR. This difference was statistically significant ( *p* -value = 0.008) ( Table 4 ).

**Table 4:** Odds of self-reported symptoms in functional versus structural dysphonia

Variable	Status	OR	95%CI	*p* -value
RSI ≥ 13 and AR ≥ 1 vs other	Unadjusted	2.33	0.704–7.755	0.166
RSI ≥ 13 and AR ≥ 1 vs other ^¥^	Adjusted	2.91	1.32–6.44	0.008*

**Abbreviations** : AR: allergic rhinitis; OR: odds ratio; RSI: Reflux Symptom Index.

**Notes** : *Statistically significant; ^¥^ Adjusted for smoking.

## Discussion

Laryngopharyngeal reflux is a significant risk factor for dysphonia. Suggested mechanisms include micro-aspiration of the refluxate material into the larynx with direct injury to the mucosal lining, and/or a defective vagally mediated reflex of the distal esophagus. 11

In addition, prolonged exposure of the posterior larynx to refluxate material has been shown to cause inter-arytenoid muscle dysfunction, leading to laryngeal compensatory stress. This stress results in increased muscle tension and altered laryngeal dynamics. These changes result in chronic voice fatigue and contribute to the development of functional dysphonia 17

The clinical presentation of LPR may be misleading as most affected patients have no symptoms of heartburn and regurgitation suggestive of typical reflux disease. When laryngeal symptoms are present, vocal fold abnormalities, structural or neurologic, may or may not be present. 18 To that end, many studies have shown a higher prevalence of LPR in patients with functional voice disorders in comparison to patients with structural and or neurologic voice disorders. 11 In one study by Hamdan et al., 58.14% ( *p* < 0.001) of patients with globus pharyngeus, which is a common symptom of LPR, had a functional voice disorder in comparison to 41.86% in patients with structural voice disorders. 19 Groenewald et al. studied a group of 51 patients diagnosed with non-organic voice disorders and noted findings suggestive of LPR on laryngeal examination in 63% of their study group, the majority of which (94%) had an abnormal RSI score. 20 Similarly, Cesari et al. reported abnormal electro-acoustic measures in LPR-indicative pH-metric parameters in patients with dysphonia in the absence of laryngeal organic pathologies. 17 In our study, 62% of patients with functional dysphonia (31/50) had positive RSI score compared with 54% of patients with structural dysphonia although the difference was not statistically significant.

Voice disorders have also been associated with respiratory allergies, particularly in patients with preexisting co-morbidities such as asthma and atopy. Krouse et al. proposed the “unified airway model” in 2008, a conceptual framework integrating the functions of the upper and lower respiratory tracts, including asthma, allergic rhinitis, and chronic rhinosinusitis. 21 Suggested mechanisms responsible for dysphonia include postnasal drip due to hypersecretion of nasal glands caused by increased inflammatory mediators in the blood as a response to a specific allergen, leading to throat clearing, chronic cough, and hoarseness, and exaggerated rhino-laryngeal reflexes resulting in increased muscle activity. This repetitive, exaggerated laryngeal response leads to altered laryngeal muscle dynamics and leads to functional dysphonia. Simberg et al. found a higher prevalence of voice symptoms in patients with confirmed allergy undergoing immunotherapy in comparison to patients with no allergy. 22 Turley et al. reported a higher prevalence of vocal symptoms in patients with AR and non-allergic rhinitis (32.8% and 26.9%, respectively), in comparison to a control group (8.1%). 4 Also, Randhawa et al. noted a higher mean Voice Handicap Index (VHI) score in patients with positive testing for 4 or more allergens than in patients with no allergy (23.7 versus 7.8, respectively). 23 Looking at the other side of the coin, Brook et al. found that half (51.8%) of patients with laryngeal symptoms tested positive for at least one inhalant allergen. 24 In the review by Altman et al., the authors reported allergy in 37% of dysphonic patients with muscle tension dysphonia. 25 Similarly, Hamdan et al. reported that 50% of patients diagnosed with primary dysphonia tested positive for at least 2 allergens, and 36% tested positive for at least 3 allergens. 26

The results of the aforementioned investigations concur that LPR and allergy are significantly associated with functional dysphonia. Studies have also shown the cross-cutting in the clinical presentation of these two entities in patients with dysphonia. In a pilot study on 15 patients with primary voice disorder, Randhawa et al. found that 3 had concomitant LPR and allergy. 27 No previous study examined the association between both LPR and allergy and the type of dysphonia. The findings of this investigation indicate a higher prevalence of both LPR and allergy in patients with functional dysphonia as compared with structural/neurologic dysphonia. Patients with history suggestive of LPR and allergy had 2.33 times the odds of having functional dysphonia compared with those with either reflux or allergy or none.

The present study is the first to explore the combined predictive value of LPR and allergy in diagnosing dysphonia. While prior research has individually linked LPR and allergy to voice disorders, no study has examined their combined significant value in differentiating functional dysphonia from structural or neurologic etiologies. Our findings suggest that patients presenting with both LPR and allergy are at a significantly higher likelihood of having functional dysphonia, which could help in more accurate diagnosis and targeted management.

The generalizability of these findings to the global population warrants careful consideration. This investigation was conducted within a specific geographic and clinical context, potentially limiting its representation of populations with varied dietary habits, allergen exposures, and healthcare access. Furthermore, cultural differences in voice usage, smoking habits, and reflux management could significantly influence the observed relationships. To validate and extend our observations, future multi-center studies are crucial.

The main limitation of the present study is its retrospective nature, which did not allow us to exclude other possible confounding factors. Another limitation is the lack of gold-standard testing of LPR, such as double-probe pH-metry, and allergy such as skin testing. That being stated, the authors need to stress that patients with dysphonia and history suggestive of LPR and allergy, not diagnosed with LPR and allergy, are more likely to have functional etiology rather than structural or neurologic etiology.

## Conclusion

In summary, the findings of this investigation imply that patients with dysphonia and history of LPR and allergy are more likely to have a functional underlying etiology. In a primary care setting where flexible or telescopic laryngeal examination is not readily available, this information helps guide the primary caring physician in deciding on the urgency for referral to an otolaryngologist for further investigation.
